# Practical Steps to Design Brucellosis Preventive Interventions: An Intervention Mapping Approach

**DOI:** 10.1002/hsr2.70369

**Published:** 2025-01-23

**Authors:** Arash Ziapour, Farshid Rezaei, Mohammad Jafarzadeh, Nasim Mirzaei, Eslam Moradi‐Asl, Nazila Nejhaddadgar

**Affiliations:** ^1^ Cardiovascular Research Center, Health Policy and Promotion Institute, Imam‐Ali Hospital Kermanshah University of Medical Sciences Kermanshah Iran; ^2^ Psychology Research Centre Khazar University Baku Azerbaijan; ^3^ Health Education and Promotion Department, Health Deputy Ministry of Health Tehran Iran; ^4^ Department of Infectious Diseases, School of Medicine Ardabil University of Medical Sciences Ardabil Iran; ^5^ Department of Public Health, Firoozabad Branch Islamic Azad University Firoozabad Iran; ^6^ Department of Public Health, School of Public Health Ardabil University of Medical Sciences Ardabil Iran

**Keywords:** brucellosis, educational programs, intervention mapping, livestock farmers, social listening

## Abstract

**Background and Aims:**

Common diseases between humans and animals are one of the health problems of countries, which requires targeted intervention. The intervention mapping model provides guidance for choosing the most appropriate methods and applications. Since one of the most important challenges in the endemic areas of Iran is the control of brucellosis. Therefore, the aim of this research is to examine the intervention mapping model and its steps to design interventions to prevent this disease.

**Methods:**

In this research, the practical steps of the Brucellosis prevention intervention were carried out with the intervention mapping approach in six steps from July 2023 to December 2023. In the first step, needs assessment was done through literature review, semi‐structured in‐depth interview and checklist. In the second step, the matrix of change objectives was designed. After selecting intervention methods based on theory and practical applications, and producing program components and materials, the intervention was implemented and evaluated.

**Results:**

Intervention mapping helped us in designing and implementing the brucellosis control educational program with the participation of participants, specifying the definition of results, performance goals and determinants, theoretical methods, practical applications, intervention design, implementation and step‐by‐step evaluation.

**Conclusion:**

The intervention mapping approach ensures the transparency of all intervention components and it provides a useful approach for developing behavior change interventions.

**Abbreviations**
PI: preventive interventions
IMA: intervention mapping approach

## 1 Introduction

Diseases common to humans and animals are now considered among the major health issues worldwide [1]. Brucellosis is one of the most common bacterial infectious diseases in humans and animals, which is directly or indirectly transmitted from an infected animal, Infected products may contaminate humans, which is a major issue for public health and veterinary medicine [2]. Consumption of unpasteurized milk, raw or semi‐raw meat and close contact with the body secretions of an infected animal are the main reasons for the transmission of this disease to humans. Are considered a major source of infection in developing countries because of the lack of the prerequisites of disease control [3, 4]. According to the World Health Organization, out of every five infected cases, only one is diagnosed [5]. In Iran, the main risk factors of Brucellosis are the consumption of nonpasteurized dairy products (especially raw milk, fresh cheese and nonpasteurized ice cream), direct contact with animals and animal husbandry, and on the other hand, the lack of knowledge and false beliefs of people about the ways of transmission of this disease. Also, the geographic border of Iran with Iraq, Pakistan and Afghanistan and the lack of precise control programs for livestock diseases in these countries is one of the risk factors of this disease [6]. In their study of 1698 patients from 30 provinces of Iran, Rostami et al. found that the average incidence of brucellosis is 29.83 per 100,000 population (55% male and 45% female) [6]. Kasiri et al. showed that the incidence of brucellosis in western Iran was 59.31 per 100,000 population (34.9% female and 65.1% male) and nearly 95.2% of human cases live in rural areas and 4.8% live in urban areas [7]. Considering that in Iran, as one of the largest breeding centers for Webs sheep in Central Asia, the brucellosis control program is done through vaccination. However, the control and eradication of brucellosis in endemic areas is a great challenge that requires systematic interventions [1]. There is research evidence that breaking the chain of disease transmission from animals to humans depends on targeted training that meets the needs of society (social listening), effective training that recognizes needs, habits and misinformation, how to change behavior and adopt positive and correct ways. This training leads to behavior change and learning the correct culture of consuming milk and other dairy products and how to deal with infected animals [8]. In addition to afflicting humans, Brucellosis causes many complications such as abortion in livestock and reduction of milk production, and economic damages to the export of dairy products. Thus, it requires intervention in different aspects, including education and culturalization based on needs (social listening) [1]. Traditional educational programs common in health systems, which originally aim to affect the target populations and improve health literacy fail to work for village residents and livestock farmers as other factors are involved in the process of spreading the disease [8]. Some experts believe that one reason for the failure of educational programs is inadequate attention to cognitive factors and development of programs with no basis in models of health literacy promotion and the role of people in educational programs [8, 9]. Researchers need not only to know it is necessary to follow a theory in their field, but should also know it is necessary to know the needs of the target population to design effective interventions [9]. It also guides the choice of the most appropriate methods and practices for the target population through with social listening [10, 11]. This approach suggests 6 steps, a needs assessment (social listening), matrix of change goals, selection of intervention methods based on theory and practices, development of components and materials of the intervention program, planning for adoption, implementation and evaluation of the intervention and, thus makes it possible to make effective decisions in each step of intervention, implementation and evaluation [11, 12]. The present study aims to examine this approach to prevent brucellosis. If used correctly, it can lead to the development and implementation of effective interventions.

## 2 Materials

The present study describes the practical steps of intervention to prevent brucellosis using an intervention mapping approach. Before beginning the design of the intervention process, a participatory planning group was formed, including provincial health officials, representatives of the group to fight diseases common to human and animal, health volunteers and influential people in neighborhoods and health experts. The aim was to ensure the participation of all stakeholders in social listening. The intervention plan was made as follows.

The criterion for entering the study was at least 6 months of work experience among health workers and health volunteers, the willingness of people to participate in interviews and group discussions, and have the ability to express their experiences. Exclusion criteria included people's unwillingness to continue cooperating in the study and lack of consent to record interviews and group discussions. To select the participants in the study, a purposeful sampling method was used with respect for diversity. The diversity of interviewees was chosen from all levels of the health system and the diversity of patients in different areas covered by the University of Medical Sciences.

### 2.1 Step 1: Needs Assessment

The first step of the intervention design was needs assessment, done through a literature review of 61 articles relevant to the topic in the past 5 years, semi‐structured in‐depth interview and a checklist. In this step, semi‐structured in‐depth interviews were held with 52 people suffering from Brucellosis in the past 1 year, 15 healthcare workers and 15 health volunteers for social listening and collecting opinions about barriers and solutions to reduce Brucellosis and its facilitators. For needs assessment, a checklist was used to assess the current condition of patients with Brucellosis based on the PRECEDE–PROCEED^1^ model. The checklist enquired about demographic variables, predisposing factors, enabling factors (environmental factors), reinforcing factors (encouragement) for a total number of 2679 people.

### 2.2 Step 2: Development of a Matrix of Change Objectives

In the second step, a matrix of change objectives (expected changes in behavior or environment) was developed according to the results of the first step. The results of the literature review were used along with the results of the checklist to know the current condition of 2679 patients with Brucellosis (Patient information is available on the website of the Ministry of Health's disease center, separately for each province. Your information, personal characteristics, high‐risk behaviors in the case of Brucellosis disease (Which unhygienic behavior has the patient had in the last month that has an effect on Brucellosis disease?). Together with a semi‐structured in‐depth interview, the most modifiable determinants of the incidence of Brucellosis in high‐incidence areas were identified to guide the promotion of healthy behavior to prevent and control the disease. The identified determinants were divided into functional objective. The last step of determining change objectives was written for each functional objective, and the determinants and functional objectives were integrated into a matrix of change objectives. Finally, a table of what needs to be done was drawn.

### 2.3 Step 3: Theoretical Methods and Practices

The third step was the selection of methods based on educational models and suggestion of practical solutions. Intervention is a process in which theory‐based methods are used to change environmental or behavioral conditions. For example, the social learning theory is selected and used as a basis for improving self‐efficacy. This step involves a selection of interventional methods or programs according to the features of target population and their culture. It was done with the help of health workers and health volunteers asked to give feedback and comment on the programs run by the planning group to better achieve the objectives of change as reflected in people's comments.

### 2.4 Step 4: Development of Program Content

This intervention step was taken by the research team, along with all components and materials needed for the development and application of standard and comprehensible educational content for people in goal‐oriented education. The pre‐test was prioritized to implement the functions of the program content to cover the objectives of change by the educational material and match the local culture. Finally, the structure and time of intervention (where and when to talk to people and give training were decided on, as all these had to be in accordance with people's conditions, not the researcher's likes or dislikes).

### 2.5 Step 5 and 6: Implementation and Assessment of Program

The intervention program was designed in 2 of the 10 counties of the province marked by the highest incidence rate in the past year. The interventions were planned rural family and general public levels. A suitable logo and slogan were designed for the program. Appropriate methods of intervention a the individual, familial and social levels were determined regarding the ways to prevent brucellosis.

## 3 Results

The analysis of the checklist of 2679 patients with brucellosis showed that the prevalence of this disease is directly related to the place of residence of people. Actually, it is distributed as a cluster in the northwest of the studied area (Ardebil province) and the prevalence of the disease is higher in rural areas. There are two important centers of the disease in the province, both located in the rural areas of the north and the center of the province. The results of IDW interpolation analysis explain that there are two hotspot regions in Ardabil in terms of the prevalence of Brucellosis. Aslanduz country is a high‐risk region in the north of the province and its risk of infection is between 13% and 15% [1]. This center is a region with traditional animal husbandry and 50% of its residents are nomads with many livestock. Also, more than 60% of patients were livestock farmers or held jobs related to livestock and transporting milk and their families. About 90% of patients had the experience of consuming unpasteurized or raw dairy products.

As the results of the first step (review of the literature) showed, these are the key factors in transmitting the disease: incomplete vaccination of animals (cows, sheep, goats), direct contact through the conjunctiva of eyes or through scratches and skin injuries with secretions and excreta or tissues of infected animals or objects covered with infectious secretions, foods or liquids containing brucella bacteria, raw milk and contaminated raw dairy products, meat, organs, respiratory transmission through inhalation of infectious particles suspended in stables, and laboratories, artificial insemination, vaccination, and blood sampling [13, 14, 15, 16, 17, 18]. In a semi‐structured in‐depth interview with people (social listening), the use of raw and unpasteurized dairy products and unhealthy behavior in livestock farmers were the two most important causes of brucellosis in two disease centers of Ardabil province.

### 3.1 Step 2: Development of the Matrix of Change Objectives

In this step, as the results of the first step showed, a draft of functional objectives was developed based on theories (Table 1). Then, for validation, the opinions of behavioral, infectious, and health and promotion experts were used to review performance objectives, a focal group method was used to select the key changeable determinants for intervention. At the individual level (knowledge, attitude, perceived susceptibility and self‐efficacy), familial and social level (subjective norms) were selected as the determinants. At the end of the matrix, the change goals or intervention roadmap was set.

**Table 1:** Objectives of change matrix.

Functional objective	Knowledge	Attitude	Self‐efficacy	Subjective norms	Behavioral barriers	Perceived susceptibility
Livestock farmers should vaccinate their animals against Brucellosis disease.	Livestock farmers should name three reasons for the significance of animal vaccination. Livestock farmers should state three consequences of not vaccinating livestock.	Livestock farmers should express their willingness to vaccinate livestock through participation and Q & As. Farmers should discuss the positive effects of livestock vaccination in meetings.	Livestock farmers should show confidence in the complete vaccination of livestock. Livestock farmers should show confidence in the required follow‐up of animal vaccination and reducing possible barriers.	Farmers should state the role of important people in accepting and not accepting livestock vaccinations. Livestock farmers should state they can get help from influential people and experts to follow up animal vaccination.	Livestock farmers, stakeholders and health officials should name the possible barriers to livestock vaccination. Livestock farmers should offer solutions to reduce the possible barriers to vaccination according to the existing conditions.	The livestock farmer should consider and express the Brucellosis disease as a threat to human and animal health. Cattle breeders should recognize brucellosis as an incurable and dangerous disease.
Livestock farmers should participate in training sessions on ways to prevent and control Brucellosis.	Farmers should state the significance of complete vaccination of livestock. Livestock farmers should state the significance of a healthy living place for livestock. Farmers should state the significance of disinfecting the place where animals are born. Livestock farmers should state the necessary health tips when they are present in a place where animals are kept while milking and touching the animals. Farmers should state the significance of washing hands regularly and taking care of skin wounds.	Livestock farmers should talk in a peer group about their experiences while being at the livestock keeping place. Livestock farmers should talk about the importance of observing health precautions while being at the place of keeping livestock in the group of peers.	Livestock farmers should be self‐confident in implementing health tips to prevent brucellosis. Farmers should be confident in their ability to participate and discuss in peer group meetings.	Farmers should say who, they think, can help and encourage them to improve and perform behavioral skills.	Cattle breeders should talk about the potential barriers to compliance with health precautions in places where animals are kept. Livestock farmers should talk about the potential barriers to compliance with health precautions in consuming pasteurized dairy products.	Livestock farmers consider nonobservance of health precautions in the keeping place of livestock as a threat to their health and livestock.
Health volunteers, general public and local stakeholders should participate in training sessions on practical ways to prevent and control Brucellosis.	Health volunteers, the general public and local stakeholders should state the significance of using pasteurized dairy products to prevent Brucellosis. Health volunteers, the general public and local stakeholders should know and follow the necessary health tips when consuming unpasteurized milk. Health volunteers, general public and local stakeholders, if consuming unpasteurized cheese, must keep the cheese in salt water for at least 3 months and then consume it. Health volunteers, general public and local stakeholders should keep the milk at the boiling point for 5 min after boiling and only then consume it if they consume unpasteurized milk.	Health volunteers, the general public, and local stakeholders discuss their experiences in a peer group. Health volunteers, the general public and local stakeholders should discuss the significance of following health tips in the peer group.	Health volunteers, general public and local stakeholders should be confident in their ability to implement health tips at home. Health volunteers, the general public and local stakeholders should be confident in their ability to participate and discuss ideas in peer group meetings and disseminate relevant health information to the public.	Health volunteers, the general public and local stakeholders should state who can contribute to the improvement of behavioral skills to prevent Brucellosis.	Health volunteers, the general public and local stakeholders should talk about potential barriers and solutions to compliance with health tips according to their existing conditions.	Health volunteers, general public and local influential people should consider nonobservance of health tips as a threat to their health and family.
Livestock farmers should attend special trainings to set up industrial animal husbandry.	Livestock farmers should state the importance of setting up industrial livestock farming.	Farmers should talk about their positive experiences	Farmers should be confident in their ability to implement industrial animal husbandry.	Livestock farmers should state who can play a significant role in setting up industrial livestock farming.	Livestock breeders should state the potential barriers to industrial animal husbandry. Cattle breeders should state the effective solutions to reduce the potential barriers to setting up industrial animal husbandry.	Livestock farmers consider failure to start industrial animal husbandry as a threat to the health of their livestock and themselves.

### 3.2 Step 3: Selection of Theoretical Methods and Practices

In the third step, as the results of the social listening showed, the use of raw and unpasteurized dairy products and the unhealthy behavior in livestock farmers were found to be two important causes of Brucellosis in Ardabil province, which were modifiable through intervention. The target population and their culture were selected, and to ensure compliance with local culture, an initial assessment was made with the help of health workers and health volunteers. After correcting the necessary items, they were selected for the final intervention (Table 2).

**Table 2:** Theoretical methods and practices for intervention.

Determinants	Relevant theory	Definition	Content	Channel and medium	Functional plan/practice
Knowledge	Consciousness raising (trans theoretical model) Risk personalization (Precaution Adoption Process Model)	Providing information, feedback on reasons, offering solutions Providing information about the individual costs of performing the behavior or not performing	Introducing reliable sources and databases, introducing barriers to the implementation and solutions to prevent Brucellosis	Healthcare givers Health volunteers and stakeholders Interview, video	Poster, motion graphics, paint motion, logotype, infographic, booklet, social listening
Attitude	direct experience (learning theory) Cultural similarity (multiplicative relationship matrix)	Encouraging a process in which knowledge is constructed through interpretation of practice Using features of the target group in the source, message	Advice and persuasion by successful farmers regarding industrial animal husbandry, explaining the benefits of vaccination	Healthcare givers	Influential people, heroes, videos, conventions and Social listening
Self‐efficacy	Offering rewards (learning theory) Goal setting (self‐regulation theory)	Encouraging or offering rewards that are clearly linked to the achievement of the target behavior. Making a list of potential barriers and ways to overcome in the presence of successful people	Encouraging people who decide to reduce the barriers to Brucellosis prevention strategies Goal‐oriented planning for vaccination and setting up industrial animal husbandry Offering creative solutions for vaccination and setting up industrial animal husbandry	Healthcare givers, interview, video	Influential people, heroes, movies, conventions and Social listening
Subjective norms	role modeling by mass communication (Diffusion of Innovation (DOI) Theory)	Proposing the right model to encourage via mass media Encouraging social networks to provide information, feelings, encouragement and support	Introducing successful people by using the capacity of influential and influential characters	Healthcare givers	Social listening and convention
Behavioral barriers	Planned coping response (theory of planned behavior)	Encouraging the individual to identify potential barriers and suggesting ways to overcome them	Introducing people who decide to reduce barriers to the prevention of Brucellosis	Healthcare givers	Social listening and convention
Perceived susceptibility	Risk personalization (Precaution Adoption Process Model)	Providing information about the individual costs or risks of activities relative to the target behavior	Introducing the consequences of nonobservance of healthy and preventive behavior	Healthcare givers, interview, video	Social listening and convention

### 3.3 Step 4: Development of Program Content

In this step, the research team decided on the scope of intervention (areas that had the highest rate of incidence) and the most suitable channels and local influencers to implement the intervention. In this step, a wide range of potential strategies were determined, so that the strategies and objectives of change in the intervention could be addressed. They were all re‐examined (for verification) and, finally, the main components of intervention were selected based on feasibility and available resources. To make sure that the intended messages and information were clear and comprehensible, the educational messages were given to 5% of livestock farmers, the general public, and influential people in each region. Occasional parts that needed to be corrected were rewritten. The intervention program was aimed for a period of 6 months.

### 3.4 Step 5 and 6: Implementation and Assessment of Program

The intervention program was designed for two cities marked by the highest incidence rate within the past year at the individual (farmer), familial (rural family) and social (general public) levels. At first, the logo and the slogan “No to nonpasteurized dairy” and “I vaccinate my animals” were developed for intervention programs. Intervening at the individual level by giving speech in the neighborhood and the workplace of the rancher and visiting health centers with motivational messages, putting up posters, publishing motion graphics on the internet (popular channel in the village) and appealing to local influential people and most importantly talking were the ways to listen to people's ideas. Interpersonal level of intervention included providing a platform for the formation of peer groups for discussion and exchange of ideas in the presence of health workers or healthcare workers. The social level of intervention included holding local festivals with the participation of successful livestock farmers and putting up banners. To evaluate the progress of the work process, health volunteers and healthcare workers were asked to have focal group discussions with livestock farmers twice a month so that they can better explore the implementation process and barriers and inform the research team. If there was a barrier to the implementation of programs, it could be identified and removed with the help of local stakeholders.

Assessment of the impact of program was done based on the general goal and specific goals. It used the completed measurement instruments in the needs assessment step. Goal of designing this intervention program: 1‐reduce the incidence rate of brucellosis in humans by 10% last year, 2‐to increase the community's knowledge of the ways of transmitting and preventing brucellosis by 20% of the base number. The specific goals included a relative increase in the number of educational content and information to livestock farmers and the general public. Due to the importance of developing an intervention program based on needs assessment and social listening, in the present study, the research team developed an intervention map. The results of the intervention, which requires at least 6 months of time, will be published in the next article which is being implemented by the same research team.

## 4 Discussion

The intervention mapping approach ensures transparency of all aspects of intervention and provides a useful approach to develop behavior change interventions. Identifying the determinants of behavior helps health planners and policy makers select appropriate behavior change strategies to have systematic plans for local and regional interventions.

Intervention mapping is now widely used in the design of interventions to prevent disease and health problems worldwide. This model has been used in various fields of health promotion programs, including the prevention of communicable and noncommunicable diseases, as well as the promotion of public health. Most of the reports related to intervention mapping indicate its usefulness in designing disease prevention programs with different severities in all types of patients.

Based on the results of the studies, increasing the information of livestock farmers (as beneficiaries in the intervention process) is an important factor in increasing their sensitivity and preventing brucellosis, which is predicted in the intervention mapping model [19].

Studies show that it is easier to change people's behavior by learning about the benefits of the behavior and the cost effectiveness. Also, paying attention to environmental factors and obstacles and people's participation in changing behavior is very effective because people consider themselves the owners of the process and will cooperate better. which is one of the most important differences between this model and other models [12, 20].

## 5 Conclusion

The intervention mapping approach ensures the transparency of all intervention components and provides a useful approach for developing behavior change interventions and the results of this study led to the formulation of an intervention program for the prevention of Maltese fever based on theory and It becomes evidence. Also, identifying the determinants of behavior helps health planners and policy makers in choosing appropriate behavior change strategies.

## 6 Study Strengths and Limitations

The limitation of the design of this intervention program is the design based on the priorities of a province, which may limit its generalizability, although the ways of disease transmission and prevention are the same in most regions, the use of the intervention mapping model in the design and formulation of health interventions by having a team of stakeholders and experts as participants in the formulation of the program is the most important step to develop a targeted program that can replace conventional programs above to be down regardless of the background of the society. Without needs assessment, social listening and knowledge of influencing factors in behavior or health outcome, in most cases the programs will be ineffective and sometimes fail, which imposes a lot of cost on the health system. The future plan of our research team is to evaluate the effectiveness of this intervention model in the implementation field. Then, using the results of that study, an intervention can be carried out on a larger scale and its generalizability can be evaluated.

## Author Contributions

**Arash Ziapour:** conceptualization, methodology, data curation, project administration, resources, supervision, validation, writing–review and editing. **Farshid Rezaei:** conceptualization, investigation, methodology, writing–original draft. **Mohammad Jafarzadeh:** data curation, formal analysis, writing–original draft**. Nasim Mirzaei:** validation, visualization, writing–review and editing. **Eslam Moradi‐Asl:** validation, investigation, writing–original draft**. Nazila Nejhaddadgar:** conceptualization, investigation, methodology, writing–original draft. All authors have read and approved the final version of the manuscript. Nazila Nejhaddadgar has full access to all of the data in this study and takes complete responsibility for the integrity of the data and the accuracy of the data analysis.

## Ethics Statement

The Ethics Committee of the Ardabil University Medical Sciences (IR.AUMS. REC.1402.196.) confirmed this study. All participants were informed of the study, and just those who signed a written informed consent form were included in the study.

## Conflicts of Interest

The authors declare no conflicts of interest.

## Transparency Statement

The lead author Nazila Nejhaddadgar affirms that this manuscript is an honest, accurate, and transparent account of the study being reported; that no important aspects of the study have been omitted; and that any discrepancies from the study as planned (and, if relevant, registered) have been explained.

## Data Availability

The datasets used in the study are available from the corresponding author on reasonable request.

## Endnotes

1 Reinforcing, and enabling constructs in educational diagnosis and evaluation policy, regulatory, and organizational constructs in educational and environmental development.
